# Lifestyle Practices, Satisfaction with Life and the Level of Perceived Stress of Polish and Foreign Medical Students Studying in Poland

**DOI:** 10.3390/ijerph17124445

**Published:** 2020-06-20

**Authors:** Michał Machul, Monika Bieniak, Justyna Chałdaś-Majdańska, Jadwiga Bąk, Agnieszka Chrzan-Rodak, Paulina Mazurek, Piotr Pawłowski, Daria Makuch-Kuśmierz, Anna Obuchowska, Adrianna Bartoszek, Katarzyna Karska, Krzysztof Jurek, Claudia Cardenas, Beata Dobrowolska

**Affiliations:** 1 Students’ Scientific Association at the Department of Nursing Development, Faculty of Health Sciences, Medical University of Lublin, 20-081 Lublin, Poland; mm.machul@gmail.com (M.M.); monika675@poczta.onet.pl (M.B.); justynachaldas@wp.pl (J.C.-M.); jadwigabak25@gmail.com (J.B.); agnieszkachrzan607@gmail.com (A.C.-R.); mazurekpaulina20@gmail.com (P.M.); pawlowskipiotr56@gmail.com (P.P.); d.makuch7@interia.pl (D.M.-K.); annaobuchowska2@gmail.com (A.O.); adriannab97@onet.eu (A.B.); 2 Department of Foreign Languages, Medical University of Lublin, 20-090 Lublin, Poland; katarzyna.karska@umlub.pl; 3 Faculty of Social Sciences, Institute of Sociology, John Paul II Catholic University, 20-950 Lublin, Poland; kjurek@interia.eu; 4 Department of Primary Care, Avalon University School of Medicine, Atlanta, GA 30308, USA; denascar6@gmail.com; 5 Department of Nursing Development, Faculty of Health Sciences; Medical University of Lublin, 20-081 Lublin, Poland

**Keywords:** lifestyle practices, Polish students, foreign students, sociodemographic characteristics, educational migration

## Abstract

The adaptation of foreign students to a new country can be complicated due to different cultural values, language barriers and the way university courses are structured. The aim of the study was to analyze the lifestyle practices, satisfaction with life and the level of perceived stress of Polish and foreign students studying various medical disciplines in Poland with regard to chosen sociodemographic characteristics. The study included 231 foreign and 213 Polish students (*n* = 444) taking different medical disciplines at the medical university in eastern Poland. Three research tools were used: Fantastic Lifestyle Questionnaire (FLQ), Satisfaction with Life Scale (SWLS), Perceived Stress Scale (PSS-10). Additionally, students’ sociodemographic data was collected. Two-factor analysis of variance (ANOVA) was performed and correlations between variables were also examined. Our research indicated that Polish students obtained higher results in FLQ than foreign students. It also demonstrated a significantly higher level of stress among Polish students in comparison to foreign students. The self-assessment of their health condition, lifestyle, and rank associated to being healthy correlated with FLQ, SWLS and PSS-10. The present research can aid the development of support programs for foreign students so that the cultural adaptation processes would more positively influence their lifestyle and an education environment.

## 1. Introduction

Educational migration, an effect of the progressing globalization process, has brought about a shift in the perceptions of national governments. Foreign students are now not only an opportunity for economic stimulation, but also an occasion to raise the prestige of education centers, schools, and universities [1,2,3]. Open borders, and access to educational programs such as the Erasmus Program, have become enticements for self-motivated students who wish to improve their educational experience or interpersonal skills at a foreign university [4].

Cooperation of scientific centers around the world improves the quality of education, offers additional options for participant experience, and allows for the exchange of information between different cultures and their customs [5,6]. Students in a foreign country gain this knowledge and skill expansion as well as improve their ability to cooperate with other people. Consequently, their creativity and openness are triggered; facilitating their readiness for acknowledging these changes and accepting the challenges they create [7].

The acculturation adaptation of students to a foreign country involves cultural and psychological changes which include acclimatizing to the customs of a given group and its socio-economic life. In turn, this affects the attitude of the individual not only towards the psychological changes of the acculturation process but also towards his/her cultural identity and his/her social behavior in interpersonal contacts [8]. Adaptation allows the individual to function better in the new community, increases his/her opportunity for better well-being and boosts the social skills that are necessary for him/her to function in a culturally complex reality [8]. According to the available literature, acculturative negative factors experienced by foreign students include discrimination, an unfamiliar educational environment, language barriers, sociocultural stressors, and acculturative lifestyle stressors. However, within acculturation models, other positive factors present in the acculturation process include social support, cognitive assessment of life changes, and/or the adoption of appropriate coping strategies. The latter has a significant impact on the degree of acculturative stress and the adaptation process [9]. Yet, the acculturation process may have both positive and negative influence on the lifestyle and health behaviors of the foreign students [10,11]. While adopting healthy behaviors serve as a protective factor in acculturation and leads to a positive impact on health; unhealthy lifestyle choices can impact a student’s health negatively. Increased acculturation can be a risk factor, especially when the wrong lifestyle in the host culture prevail when compared to the student’s culture of origin. When the opposite is true, acculturation can serve as a protective factor [11].

For the purposes of the present paper we presume that lifestyle is understood as people choices regarding their behaviors. In the context of health, these choices include among others food, stimulants, and physical activity [12]. Interestingly, since the advent of the concept of Lalonde fields in the 1970s, the interest in the lifestyle and its relationship with health has increased. It was found that lifestyle is the largest and the direct determinant for the health of an individual. Consequently, this resulted in numerous studies discussing the impact of the lifestyle practices and health of an individual. Additionally, a number of programs aiding the maintenance of favorable health behaviors as well as the prevention of chronic, civilization-related diseases was designed [13].

The available literature indicates that studying in a foreign country affects students’ lifestyle practices. It has been reported that one of the changes influences the eating habits. The irregular and odd times of meals and a decrease in the food variety and quality when compared to the country of origin causes weight fluctuation [11,14,15,16,17,18,19,20]. A study conducted by Yan and FitzPatric [11] also indicated that foreign students in the USA reported difficulties finding healthy food on campus and therefore it required an additional effort on their part to keep a healthy diet. However, it is not only the eating habits, but also the physical activity of the students, which is affected when studying abroad. Interestingly, in the case of physical activity, students reported positive effects that is their physical activity increased after arriving to a new country [11].

Nevertheless, some authors observed an inclination for risky behaviors among foreign students. Angelin and colleagues [21] reported risk behaviors concerning sexual activity and alcohol consumption among Swedish students studying abroad. Similar results were obtained by Marcantonio et al. [22] among American students and by Aresi et al. [23] among European students while studying abroad. Moreover, it has been indicated that students are more likely to experiment with new behaviors in foreign country, regardless of their cultural beliefs [11,22]. Stress and negative emotions also prove to be the predisposing factors for negative health behaviors among foreign students [24].

The available statistics indicated a growing number of foreigners willing to study in Poland. The implementation of the “Study in Poland” program and individual actions taken by different universities resulted in an increase of 5.63% in the internationalization rate in the academic year 2017/2018. According to the data from the above-mentioned academic years, 72,743 foreign students from 170 countries studied in Poland. The main group consisted of students from Ukraine (37,829) and Belarus (6044), followed by India (2987), Spain (1889), Sweden (1160), Norway (1466), Turkey (1807), Czech Republic (1448) and Germany (1257). The number of students from Asia, for example, Taiwan (853) also increased [25]. The factors contributing to the increase of the number of students who decided to study in Poland are numerous and include the relatively low cost of living, few cultural differences, the opportunity to study in English and the recommendation or family members. The surveyed foreign students also mentioned problems they faced when they moved to Poland, that is language barrier, adverse weather conditions impacting certain activities and few cultural events in English [26].

Together with the increasing number of foreign students in Poland, the academic communities face new challenges and diverse needs of foreign students as well. The research on the changes affecting foreign students can contribute to the design of health promotion programs targeted specifically for educational migrants. There are numerous studies addressing the issues of lifestyle and health behaviors among foreign students representing different cultures and countries of origin [15,27,28,29]. The research previously conducted in Poland, focused mainly on the lifestyle and health behaviors of students from Vietnam, Taiwan and Norway [30,31,32]. However, there are very few comparative studies focusing on the lifestyle of foreign students and Polish students studying medical disciplines in Poland. Therefore, the aim of the present paper was to analyze the lifestyle practices, satisfaction with life and the level of perceived stress of Polish and foreign students studying medical disciplines in Poland with regard to chosen sociodemographic characteristics.

## 2. Materials and Methods

### 2.1. Study Design

A cross-sectional survey was conducted on a convenience sample of 444 students attending different medical courses at one accessible medical university in eastern Poland. The study was reported according to the STrengthening the Reporting of OBservational studies in Epidemiology studies [33].

### 2.2. Participants

The following is the research inclusion criteria: attending available medical or health science disciplines (like medicine, dentistry, nursing, midwifery and public health, at one accessible medical university, and the consent to participate in the study. The exclusion criteria were the lack of a consent to take part in a study and studying at faculty different than medical.

All the respondents with incomplete data were removed from the analysis. Finally, two groups of students participated in the study: 213 Polish and 231 foreign students, which comprised of 22 different countries: 39 (17.8%) from Europe, 15 (6.8%) from North America, and 165 (75.3%) from Asia. Female students constituted 70% of the respondents (n = 311), more in Polish group (182; 85.4%) than in foreign (129; 55.8%). The average age of the surveyed students was 21.9 (SD = 3.98).

### 2.3. Ethical Issues

Prior to the research, the positive consent of the Bioethics Committee at the Medical University of Lublin was obtained: KE-0254/24/2018. The study was performed in accordance with the principles of the Helsinki Declaration of the World Medical Association. All respondents were informed about the purpose of the study, signed the consent and their participation was voluntary and anonymous. In case of any doubts they were provided with comprehensive explanation.

### 2.4. Research Instruments

The following research tools were applied:

(1) Fantastic Lifestyle Questionnaire (FLQ) [34,35] examines important lifestyle practices of respondents from the last month divided into nine groups, i.e.,: F—family and friends, A—activity, N—nutrition, T—nicotine and stimulants, A—alcohol, S—sleep, seatbelts and stress, T—type personality, I—perception and insight, and C—career/social roles. Each group contains from two to four questions, and the score depends on the respondent answers and ranged from 0 to 2 points. The Cronbach’s alpha coefficient for the original English version of the scale was 0.88 [34,35]. The FLQ total score was computed as the sum of all items.
(2) Satisfaction with Life Scale (SWLS) contains five statements regarding perceived satisfaction with one’s life, which can be scored from 1 to 7, where 1 indicates definite disagreement, and 7 indicates definite agreement with the provided statement. The Cronbach’s alpha coefficient for the original English version of the scale was 0.87 [36]. The SWLS total score was calculated as the sum of all five items.
(3) Perceived Stress Scale (PSS-10) includes 10 questions dealing with perceived stress during the previous month. Each question should indicate how often the respondent was thinking and feeling in a similar way and the answer ranged from 0 to 4 points, where 0 indicates *never* and 4 indicates *I was thinking or feeling in a similar way very often*. The Cronbach alpha coefficient for the original English version of the scale ranged from 0.84 to 0.86 [37]. The PSS-10 total score was computed as the sum of all items.

Additionally, students’ sociodemographic data was collected and it included age, gender, marital status, county of origin, financial situation, self-assessment of health condition, self-assessment of lifestyle, change in their lifestyle while at university, as well as the importance of being healthy.

### 2.5. Data Collection Process

The research was conducted from May 2018 to January 2019. The paper and pencil method were implemented, copies of questionnaires were distributed among participating students by our research team. The team consists of students who were members of the Scientific Association, and had previously undergone a training on data gathering process. All the survey participants were explained the aims of the study and the procedure of data collection. The participants of the study were approached using the snowball sampling method [38]. One student indicated another one who was willing to participate in the study, who was contacted by the research team. A total number of 600 questionnaires were distributed among students and 485 were returned (81%). The questionnaire completeness check allowed for the inclusion of 444 surveys into the analysis. Foreign students received the original English version of the research questionnaire (already validated on English speaking populations), while Polish students received the same questionnaires validated previously in Polish culture, thus available in Polish language.

### 2.6. Analysis

The analysis was performed using the IBM SPSS Statistics version 25 package (IBM, Krakow, Poland). Two-factor analysis of variance (ANOVA) in the 2 × 2 intergroup scheme was performed in order to influence two classifying factors on the values of the analyzed variables. Correlations between variables were also examined. The level of significance (alpha) in the analysis was *p* < 0.05.

## 3. Results

### 3.1. Descriptive Statistics of Polish and Foreign Students for the Tested Variables

The means, standard deviations and reliabilities of measures were examined, with the results presented in Table 1.

### 3.2. Association of Lifestyle Practices, Life Satisfaction and the Level of Perceived Stress in Polish and in Foreign Students with Chosen Sociodemographic Characteristics

A two-factor analysis of variance was performed in a 2 × 2 intergroup scheme. The factors that were taken into consideration included the following: nationality (Polish vs. foreign students) and gender (female vs. male). The dependent variables were, FLQ, SWLS and PSS-10. For FLQ and PSS-10, the main effect of nationality was obtained (successively F (1.439) = 8.520; *p* = 0.004; eta squared = 0.019, small effect size; F (1.439) = 17.039; *p* < 0.001; eta squared = 0.037, small effect size). 

**Figure 1:**
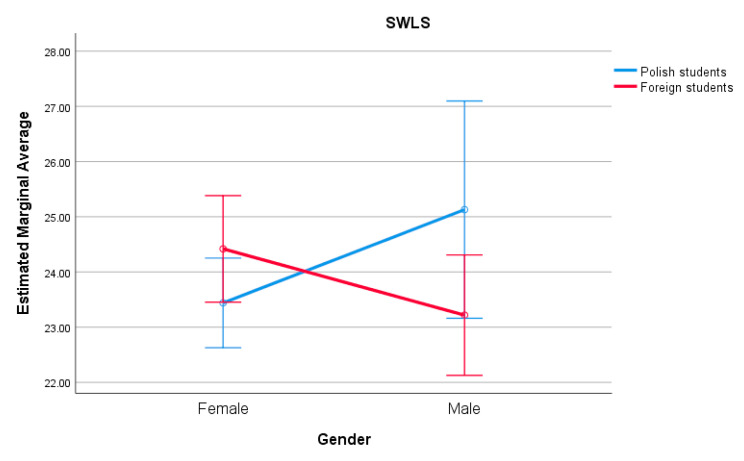
Means for SWLS by gender and nationality.

Polish students obtained higher scores on the FLQ scale than foreign students. Also on the PSS-10 scale, Polish students obtained higher results than their foreign colleagues. Main effects of gender and interaction effects were not statistically significant. Such an interaction effect was statistically significant in the case of SWLS (F (1.439) = 8.520; *p* = 0.028; eta squared = 0.011, small effect size) [39]. In the Polish students group, women reported lower life satisfaction than men. In the group of foreign students, men showed lower life satisfaction than women (Figure 1 and Supplementary Materials).

Correlations between variables were also examined. It was found that self-assessment of health condition, self-assessment of lifestyle, and the rank assigned to being healthy were positively related to lifestyle practices in both groups of students. Additionally, the financial situation of students, self-assessment of their health condition, self-assessment of lifestyle, and the rank assigned to being healthy were positively related to satisfaction with life. A level of perceived stress was negatively related to change of students’ lifestyle while at university (Table 2 and Supplementary Materials).

## 4. Discussion

The aim of this study was to compare the lifestyle practices, satisfaction with life and the level of perceived stress among Polish and foreign students studying medical disciplines in Poland with regard to the variables described among others by self-assessed health condition, lifestyle and value of being healthy.

Our study revealed that Polish students scored higher in FLQ than foreign students. Overall, the research conducted by other authors confirmed that foreign students had to struggle more to preserve a healthy lifestyle when compared to Polish students. This challenge mostly regarded the eating habits and other choices made by foreign student [11,14,15,16,17,18,20]. Moreover, some authors found that foreign students were more likely to undergo risky health behaviors. For example, study conducted by Zalewska-Puchała and colleagues [32] proved that foreign male students from Taiwan, studying in Poland, were more frequently exposed to the use of cigarettes and alcohol. It was noted that a students’ stay in another country, brought on poorer lifestyle choices regarding their health. In the study of Lolokote et al. [40], Chinese students had significantly higher results than the foreign colleagues in the average total results of the Health-promoting lifestyle-II Scale and for each of the subscales (physiological health, psychological health, societal health), which suggests that foreign medical students were less often involved in health-promoting behaviors. Similarly, Carpenter and co-authors [41] also have shown that foreign students frequently find it difficult to adapt to the new reality of a foreign country. This is mainly attributed to such factors as feeling homesick, difficulty in building new relationships, unknown diet, language barrier, differences in the education system, religious differences, health or financial problems [40].

Our study showed that both Polish and foreign students who assessed their health condition higher, those who assessed their lifestyle healthier and those who ranked the importance of being healthy better achieved significantly higher results on FLQ. Importantly, in the case of foreign students, also their financial situation correlated with better results on FLQ. What is more, studies performed by other authors confirmed that the financial situation for participants is linked with the analyzed lifestyles related to health. Additionally, according to these studies also other sociodemographic variables correlated with students’ health related practices, e.g., gender, age and marital status [16,20,28,42].

The present research indicated that the life satisfaction of foreign and Polish students tended to be on the same level. The difference appeared between gender of students. Polish female students and foreign male students obtained higher results in SWLS. On the contrary, different results were detected by Pan et al. [43], in a study among Chinese students studying in Australia. Here, foreign female students showed a higher level of life satisfaction than men. Skromanis et al. [28] in their study conducted in Australia among foreign and Australian students, indicated that foreign male students had lower levels of life satisfaction. Interestingly, the research conducted in 2015 among young people in Hungary, Poland and Ukraine showed, that respondents from these three countries were satisfied with their life; however, the Polish students obtained the poorest results [44]. Similarly, Sprynska et al. [45] indicated higher results of life satisfaction of students from Ukraine when compared to Polish students. What is important, the present study showed that the financial situation had a significant impact on the level of the life satisfaction of both Polish and foreign students, which is in line with other studies [46]. In our study, we did not measure the level of cultural adaptation of foreign students, which has been found associated with students’ satisfaction with life as it is observed in a study conducted by Chen and colleagues [47] among Tibetan students studying in China.

The present study indicated that the students from Poland presented a higher level of perceived stress than students from abroad. Importantly, our results do not correspond with the results of other authors who proved that foreign students were prone to more stress, anxiety and depression because of the additional challenges they were facing in different country and culture [17,24,43,48,49]. The available research also showed that Asian students more frequently experienced linguistic difficulties than European students, therefore they were more likely to struggle with establishing new relationships. Thus, language difficulties constituted a significant stress factor for foreign students [27,50,51,52]. This result corresponds with our findings, which showed that higher levels of stress was perceived by those students (native and foreign) who experienced greater impact of students’ life on their lifestyle. However, in research conducted in Poland by Klimczak and Majda, among foreign students, the results showed that the vast majority of students experience an average level of stress [30].

Additionally, our study revealed that lower stress was perceived by those foreign students who assessed their financial situation better and by those who rated higher their health condition, lifestyle, and importance of being healthy. This information can be a useful source in planning support programs for foreign students in the future and provide health promoting interventions for these students.

### Study Limitations

Our study had several limitations. One of the limitations was the relatively small number of students from Poland and abroad, and an insufficient number of diverse groups in terms of culture of origin. Additionally, the surveyed groups were not equal in terms of some demographic characteristics like gender and very heterogenous in terms of country of origin (in case of foreign students). The selection of the sample was non-random, and participants were selected because of their convenient accessibility and proximity—convenience sampling methodology. Furthermore, collected material included mostly subjective data, based on the students’ self-assessment of health condition, lifestyle and other factors, which would possibly change in a different circumstance or different setting and environment. We did not collect or analyze other important factors like students’ health problems, use of health services, length of stay in Poland and the level of cultural adaptation. Lastly, the data used in the study was obtained from students of one medical university, which can be seen as a selection bias.

## 5. Conclusions

Based on the subjective data collected from students, our research showed differences between cultures regarding lifestyle practices and the level of perceived stress. The health-related lifestyle of foreign students was revealed to be worse than that observed in the Polish students group and the reverse is true when it comes to the level of perceived stress, what may seem surprising considering the process of acculturation of international students reported by many scholars. However, this should be analyzed together with our other result, which showed that higher stress was perceived by those students who observed greater impact of students’ life on their lifestyle, which was found in both student groups.

Our results may aid the design and development of culturally sensitive university strategies that can assist foreign students in the process of adapting to a different culture so that healthy lifestyle choices are made, and a healthy educational environment is created. A qualitative study is recommended for future research studies, which would focus on factors conditioning students’ lifestyles, satisfaction with life, and the level of perceived stress. This study would potentially help identify the adaptation strategies of students and help promote healthy interventions.

## Figures and Tables

**Table 1:** Descriptive statistics.

Variables	Polish Students			Foreign Students		
	M	SD	Alpha	M	SD	Alpha
Age	22.10	4.46	n/a	21.69	3.48	n/a
Material situation (^a^)	3.28	0.50	n/a	3.15	0.55	n/a
Self-Assessment of health condition (^a^)	4.03	0.65	n/a	3.67	0.80	n/a
Self-Assessment of style of life (^b^)	3.61	0.82	n/a	3.55	0.88	n/a
Students life has changed my lifestyle (^c^)	3.17	0.74	n/a	3.45	0.69	n/a
Importance of to be healthy (^d^)	9.16	1.29	n/a	8.78	1.60	n/a
FLQ	36.26	6.21	0.78	33.55	6.71	0.80
SWLS	23.69	5.12	0.84	23.89	5.99	0.82
PSS-10	22.78	3.87	0.86	20.34	5.88	0.76

(^a^) 1-bad, 4-very good; (^b^) 1-definitely unhealthy, 5-definitely healthy; (^c^) 1-defnitely no, 4-definitely yes; (^d^) scale 1–10; FLQ—Fantastic Lifestyle Questionnaire; SWLS—Satisfaction with Life Scale; PSS-10—Perceived Stress Scale; M—Mean value; SD—Standard deviation; Alpha—Cronbach Alpha Coefficient; n/a—not applicable.

**Table 2:** Students’ self-assessment regarding health and lifestyle vs. FLQ, SWLS and PSS-10.

Variables	Polish Students			Foreign Students		
	FLQ	SWLS	PSS-10	FLQ	SWLS	PSS-10
Material situation (^a^)	0.04	0.25 ***	−0.00	0.35 ***	0.37 ***	−0.14 *
Self-Assessment of health condition (^a^)	0.35 ***	0.38 ***	−0.10	0.38 ***	0.39 ***	−0.17 **
Self-Assessment of style of life (^b^)	0.55 ***	0.37 ***	−0.11	0.40 ***	0.31 ***	−0.20 **
Students life has changed my lifestyle (^c^)	−0.09	0.04	0.20 **	−0.04	−0.01	0.13 *
Importance of to be healthy(^d^)	0.44 ***	0.26 ***	−0.11	0.25 ***	0.17 **	−0.16 *

(^a^) 1-bad, 4-very good; (^b^) 1-definitely unhealthy, 5-definitely healthy; (^c^) 1-defnitely no, 4-definitely yes; (^d^) scale 1-10; FLQ—Fantastic Lifestyle Questionnaire; SWLS—Satisfaction with Life Scale; PSS-10—Perceived Stress Scale. Pearson’s r factor was employed; * Significant at the 0.05 level; ** Significant at the 0.01 level; *** Significant at the 0.001 level.

## Supplementary Materials

The following are available online at https://www.mdpi.com/1660-4601/17/12/4445/s1, Data supplemental to the main text.
