# Supplementary material for: Lifestyle Practices, Satisfaction with Life and the Level of Perceived Stress of Polish and Foreign Medical Students Studying in Poland
Source: Int J Environ Res Public Health. 2020 Jun 20;17(12):4445. doi: 10.3390/ijerph17124445 (PMC7345250; doi:10.3390/ijerph17124445)
Supplement: Supplementary file 1 [file ijerph-17-04445-s001.pdf]

Data for the article on: **Lifestyle practices, satisfaction with life and the level of perceived stress of Polish and foreign medical students studying in Poland**

		Global FLQ	SWLS	PSS
N	Valid	444	444	444
	Missing data	0	0	0
M		34.8491	23.7928	21.5090
Me		35.0000	25.0000	22.0000
SD		6.60885	5.58273	5.16040
Minimum		5.00	5.00	.00
Maximum		50.00	35.00	40.00

_PL_foreigners			Global FLQ	SWLS	PSS
Poland	N	Valid	213	213	213
		Missing data	0	0	0
	M		36.2629	23.6854	22.7793
	Me		37.0000	24.0000	23.0000
	SD		6.20684	5.11653	3.87215
	Minimum		12.00	7.00	11.00
	Maximum		50.00	35.00	34.00
Foreigners	N	Valid	231	231	231
		Missing data	0	0	0
	M		33.5455	23.8918	20.3377
	Me		34.0000	25.0000	21.0000
	SD		6.71250	5.98995	5.88205
	Minimum		5.00	5.00	.00
	Maximum		50.00	35.00	40.00

TOTAL

	M	Me	SD	Min	Max
**Age**	21,89	21,00	3,98	17,00	50,00
**Material situation**	3,21	3,00	0,53	1,00	4,00
**Self-Assessment of health condition**	3,85	4,00	0,75	1,00	5,00
**Self-Assessment of style of life**	3,58	4,00	0,85	1,00	5,00
**Students life has changed my lifestyle**	3,32	3,00	0,73	1,00	4,00
**Importance of to be healthy**	8,96	10,00	1,47	1,00	10,00

PL

**Age**	22,10	21,00	4,46	19,00	50,00
**Material situation**	3,28	3,00	0,50	1,00	4,00
**Self-Assessment of health condition**	4,03	4,00	0,65	2,00	5,00
**Self-Assessment of style of life**	3,61	4,00	0,82	1,00	5,00
**Students life has changed my lifestyle**	3,17	3,00	0,74	1,00	4,00
**Importance of to be healthy**	9,16	10,00	1,29	2,00	10,00

FOREIGNERS

**Age**	21,69	21,00	3,48	17,00	36,00
**Material situation**	3,15	3,00	0,55	1,00	4,00
**Self-Assessment of health condition**	3,67	4,00	0,80	1,00	5,00
**Self-Assessment of style of life**	3,55	4,00	0,88	1,00	5,00
**Students life has changed my lifestyle**	3,45	4,00	0,69	1,00	4,00
**Importance of to be healthy**	8,78	9,00	1,60	1,00	10,00

Correlations

TOTAL

	**SWLS**		**PSS-10**		**FLQ**	
**Age**	-0,045	0,349	0,080	0,093	-0,007	0,881
**Material situation**	**0,321**	**<0,001**	-0,061	0,202	**0,239**	**<0,001**
**Self-Assessment of health condition**	**0,376**	**<0,001**	-0,085	0,075	**0,404**	**<0,001**
**Self-Assessment of style of life**	**0,335**	**<0,001**	**-0,155**	**0,001**	**0,470**	**<0,001**
**Students life has changed my lifestyle**	0,015	0,753	**0,103**	**0,030**	**-0,107**	**0,025**
**Importance of to be healthy**	**0,203**	**<0,001**	**-0,109**	**0,023**	**0,349**	**<0,001**

PL

	**SWLS**		**PSS-10**		FLQ	
**Age**	-0,067	0,329	0,130	0,059	0,025	0,714
**Material situation**	**0,258**	**<0,001**	-0,002	0,979	0,041	0,548
**Self-Assessment of health condition**	**0,381**	**<0,001**	-0,109	0,113	**0,358**	**<0,001**
**Self-Assessment of style of life**	**0,371**	**<0,001**	-0,111	0,107	**0,555**	**<0,001**
**Students life has changed my lifestyle**	0,043	0,534	**0,207**	**0,002**	-0,092	0,181
**Importance of to be healthy**	**0,260**	**<0,001**	-0,116	0,091	**0,441**	**<0,001**

Foreigners

	**SWLS**		**PSS-10**		FLQ	
**Age**	-0,022	0,738	0,030	0,653	-0,067	0,318
**Material situation**	**0,376**	**<0,001**	**-0,149**	**0,025**	**0,358**	**<0,001**
**Self-Assessment of health condition**	**0,398**	**<0,001**	**-0,173**	**0,009**	**0,385**	**<0,001**

**Self-Assessment of style of life**	**0,310**	**<0,001**	**-0,204**	**0,002**	**0,408**	**<0,001**
**Students life has changed my lifestyle**	-0,013	0,844	**0,130**	**0,049**	-0,048	0,473
**Importance of to be healthy**	**0,173**	**0,009**	**-0,160**	**0,016**	**0,256**	**<0,001**

ANOVA 2x2

**Dependent variable: Fantastic Life**						**Eta**
**Source**	**SS**	**df**	**MS**	**F**	**p**	
Model corrected	868.652	3	289.551	6.901	.000	.045
Constant	346779.961	1	346779.961	8265.412	.000	.950
Gender	68.570	1	68.570	1.634	.202	.004
Nationality	357.454	1	357.454	**8.520**	**.004**	**.019**
Gender * Nationality	13.222	1	13.222	.315	.575	.001
Błąd	18418.490	439	41.956			
Total	557840.000	443				
Total corrected	19287.142	442				

**Dependent variable: SWLS**						**Eta**
**Source**	**SS**	**df**	**MS**	**F**	**p**	
Model corrected	161.972	3	53.991	1.737	.159	.012
Constant	167048.506	1	167048.506	5374.475	.000	.924
Gender	4.310	1	4.310	.139	.710	.000
Nationality	15.683	1	15.683	.505	.478	.001
Gender * Nationality	150.772	1	150.772	**4.851**	**.028**	**.011**
Błąd	13644.922	439	31.082			
Total	264578.000	443				
Total corrected	13806.894	442				

**Dependent variable: PSS-10**						**Eta**
**Source**	**SS**	**df**	**MS**	**F**	**p**	
Model corrected	839.619	3	279,873	11,219	0,000	0,071
Constant	132623,012	1	132623,012	5316,486	0,000	0,924
Gender	79,947	1	79,947	**3,205**	**0,074**	**0,007**
Nationality	425,046	1	425,046	**17,039**	**0,000**	**0,037**
Gender * Nationality	32,289	1	32,289	**1,294**	**0,256**	**0,003**
Błąd	10951,125	439	24,946			
Total	216632,000	443				
Total corrected	11790,745	442				

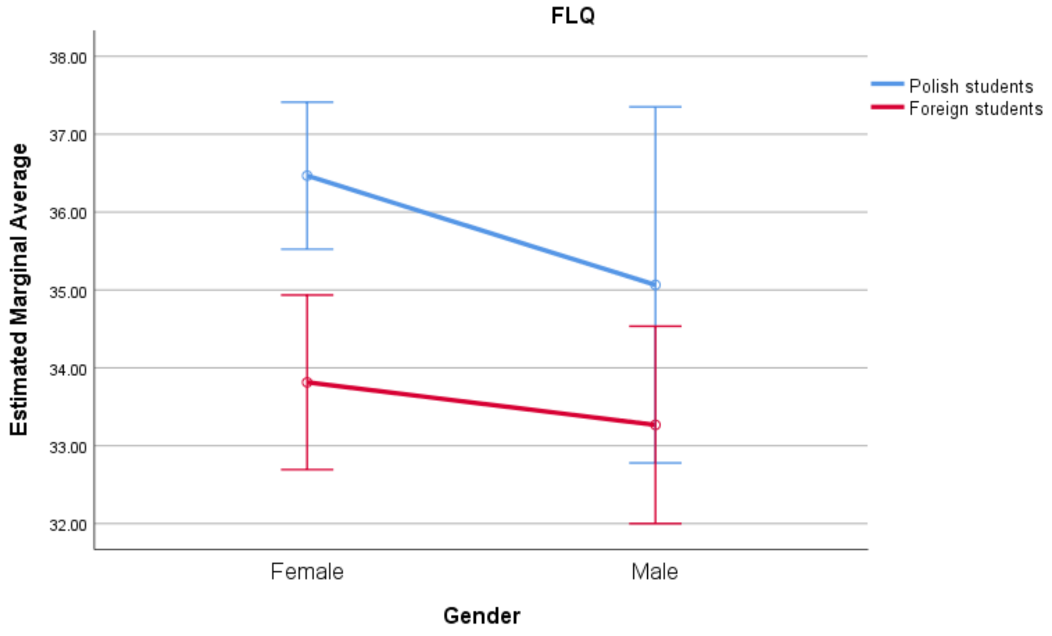
FLQ
Estimated Marginal Average
38.00
37.00
36.00
35.00
34.00
33.00
32.00
Female
Male
Gender
Polish students
Foreign students

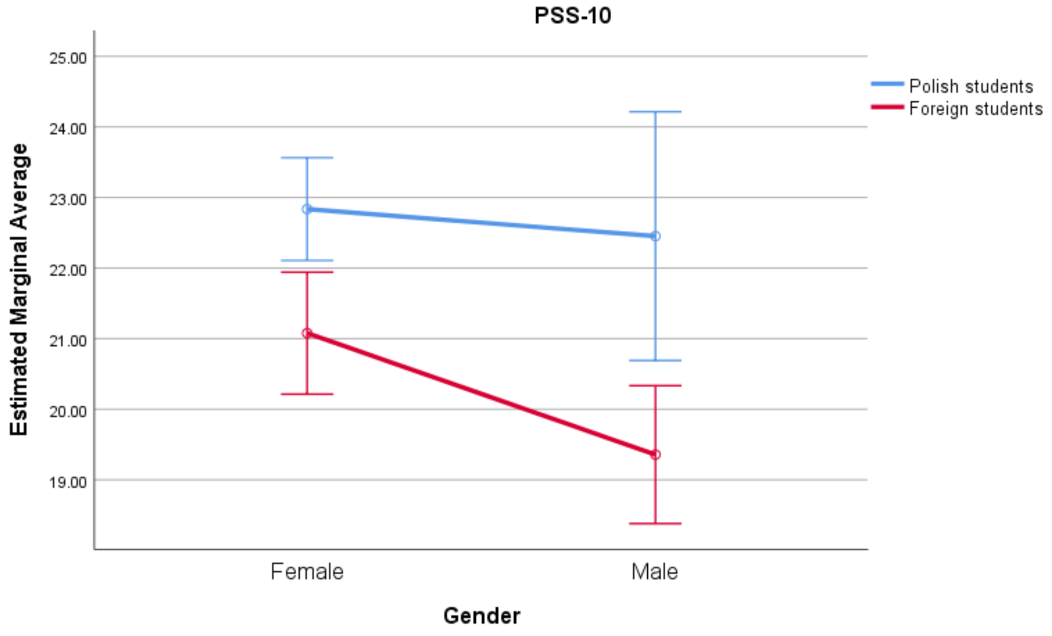
PSS-10
Estimated Marginal Average
25.00
24.00
23.00
22.00
21.00
20.00
19.00
Female
Male
Gender
Polish students
Foreign students

Multiple comparisons

Dependent variable:

Tukey Test HSD

(I) VAR00001		Difference of means (I-J)	Standard error	Significance	95% confidence interval Lower limit	upper limit
Poland	Asia	-0,17662	0,57230	0,990	-1,6525	1,2993
	America	-0,37705	1,45179	0,994	-4,1210	3,3669
	Europe	-0,26577	0,95515	0,992	-2,7290	2,1974
Asia	Poland	0,17662	0,57230	0,990	-1,2993	1,6525
	America	-0,20043	1,46311	0,999	-3,9736	3,5728
	Europe	-0,08915	0,97227	1,000	-2,5965	2,4182
America	Poland	0,37705	1,45179	0,994	-3,3669	4,1210
	Asia	0,20043	1,46311	0,999	-3,5728	3,9736
	Europe	0,11128	1,65090	1,000	-4,1462	4,3688
Europe	Poland	0,26577	0,95515	0,992	-2,1974	2,7290
	Asia	0,08915	0,97227	1,000	-2,4182	2,5965
	America	-0,11128	1,65090	1,000	-4,3688	4,1462

Dependent variable:

Tukey Test HSD

(I) VAR00001		Difference of means (I-J)	Standard error	Significance	95% confidence interval Lower limit	upper limit
Poland	Asia	2.29658*	0,51355	0,000	0,9722	3,6210
	America	2,27934	1,30276	0,299	-1,0803	5,6390
	Europe	3.12081*	0,85710	0,002	0,9105	5,3312
Asia	Poland	-2.29658*	0,51355	0,000	-3,6210	-0,9722
	America	-0,01724	1,31292	1,000	-3,4031	3,3686
	Europe	0,82422	0,87246	0,781	-1,4258	3,0742
America	Poland	-2,27934	1,30276	0,299	-5,6390	1,0803
	Asia	0,01724	1,31292	1,000	-3,3686	3,4031
	Europe	0,84146	1,48143	0,942	-2,9790	4,6619
Europe	Poland	-3.12081*	0,85710	0,002	-5,3312	-0,9105
	Asia	-0,82422	0,87246	0,781	-3,0742	1,4258
	America	-0,84146	1,48143	0,942	-4,6619	2,9790

* The difference of means is significant at the level of 0.05.

Dependent variable:

Tukey Test HSD

(I) VAR00001		Difference of means (I-J)	Standard error	Significance	95% confidence interval Lower limit	upper limit
Poland	Asia	2.55601*	0,66272	0,001	0,8469	4,2651
	America	3,70041	1,68115	0,125	-0,6351	8,0359
	Europe	3.01901*	1,10605	0,033	0,1666	5,8714
Asia	Poland	-2.55601*	0,66272	0,001	-4,2651	-0,8469
	America	1,14440	1,69427	0,906	-3,2249	5,5137
	Europe	0,46299	1,12588	0,977	-2,4405	3,3665
America	Poland	-3,70041	1,68115	0,125	-8,0359	0,6351
	Asia	-1,14440	1,69427	0,906	-5,5137	3,2249
	Europe	-0,68140	1,91172	0,984	-5,6115	4,2487
Europe	Poland	-3.01901*	1,10605	0,033	-5,8714	-0,1666
	Asia	-0,46299	1,12588	0,977	-3,3665	2,4405
	America	0,68140	1,91172	0,984	-4,2487	5,6115

* The difference of means is significant at the level of 0.05.
